# Target Elimination-Denatured and Unstable Proteins, Environmental Toxins, Metabolic Wastes, Immunosuppressive Factors and Chronic Inflammatory Factors of Medical System for Chronic Diseases Prevention and Health Promotion: A Narrative Review

**Published:** 2019-06

**Authors:** Jiren ZHANG, Zhaojing JIANG, Wenjuan WEI, Xufeng LI, Chen SUN, Yuanyuan ZHANG, Shilin FU, Jingfen ZHENG

**Affiliations:** 1. Guangdong Institute of Target Tumor Intervention and Prevention, Qingyuan, China; 2. Department of Radiation Oncology, Zhujiang Hospital, Sourthern Medical University, Guangzhou, China; 3. Institute of Economics, School of Social Sciences, Tsinghua University, Beijing, China

**Keywords:** TE-PEMIC, Prevention, Chronic diseases

## Abstract

**Background:**

The incidence of chronic diseases, such as cardiovascular disease, diabetes, overweight, obesity, cancer and other diseases has been increasing. It is a huge challenge to public health industry about how to provide risk intervention and preventive medical services and explore advanced technology platform for effective prevention and control of chronic diseases.

**Methods:**

We collaborated domestic and international experts on preventive medicine, and analyzed pathogenesis and risk factors for the major chronic diseases.

**Results:**

We established Target Elimination--denatured and unstable proteins, environmental toxins, metabolic wastes, immunosuppressive factors and chronic inflammatory factor (TE-PEMIC) system that offer us the standard and methods to eliminate and intervene pathogenic factors of the chronic diseases.

**Conclusion:**

It provides new researches and exploring new ideas to prevent and intervene chronic diseases by applying the TE-PEMIC chronic diseases prevention medical technology system.

## Introduction

“Preventive treatment of disease” is the essence of Traditional Chinese Medicine for thousands of years. With the progress of modern environmental medicine, function medicine, cellular and molecular biology, especially with the advanced technology of genomics, proteomics and metabolomics, modern medicine has entered molecular medicine, which make it possible to provide early warning of chronic diseases. Elimination of pathogenic factors and anti-aging is becoming a hot spot in health industry, which are associated with chronic diseases prevention. Prevention and intervention of in vivo exposed and residual environmental toxins (heavy metals, pesticides, toxic chemicals and microorganisms) (1), denatured and unstable proteins (proteins damaged by residue of heavy metals, pesticides, and other physical, chemical and biological factors), accumulated endogenous metabolites and toxins (increased LDL, uric acid, creatinine, HCY, Free radical, etc.), immunosuppressive factors (immune complex, Autoimmune disease related factors, autoantibody, COX2, etc.), chronic inflammatory factors (TNF-α, IL-1β, IL-6, IL-8, etc.) and imbalanced gut microbiota have important significance in chronic diseases prevention and risk intervention (2,3). Therefore, we established Target Elimination--denatured and unstable proteins, environmental toxins, metabolic wastes, immunosuppressive factors and chronic inflammatory factor (TE-PEMIC), which is a system for the prevention and treatment of chronic diseases.

### Human environmental toxins exposure and residual can cause cellular damage

With the aggravation of environmental pollution, heavy metals enter human body through a variety of ways (1). This not only damages the body’s blood vessels, nerve, immune systems, causes hematopoietic disorder, but also increases diseases incidence (2, 3). Heavy metal pollution is particularly prominent. Accumulation of heavy metals in body is closely related to chronic diseases, such as cancer. Classified arsenic, cadmium, chromium, nickel and other heavy metals are carcinogenic to human (4). Heavy metal exposure can affect human metabolism system, leading to metabolic disorders (5).The contents of heavy metal Lithium and cadmium in blood was significantly related to metabolic syndromes. Heavy metal can be detected in patients with atherosclerosis (6, 7).

Environmental risk factors can induce abnormal mitochondrial metabolism in cells to cause cardiac and renal dysfunction, ultimately lead to irreversible pathological changes (8). Long term exposure to low doses of heavy metals may cause diabetes or high blood pressure which cause occurrence and development of cardiovascular diseases (9). Exposure to heavy metals in human body may affect oxidative stress, cell cycle regulation, proliferation, methylation and DNA repair activity (10). Exposure to cadmium may lead to breast cancer, chromium and nickel exposure may increase the risk of pancreatic cancer (11), palladium and mercury exposure could increase the risk of cancerous goiter (12). Besides, comparative study between population in volcanic area and other areas shows that contents of heavy metals in water resources in the volcanic area is significantly higher than those in other areas which leads to higher incidence of thyroid cancer (13). Prevention and control of pathogenic heavy metals have been included in environment planning and remediation all over the world.

In recent years, extensive use of pesticides and insecticides has brought many negative effects. Studies suggest that long-term low dose of pesticides and pesticides residues can damage the structure and function of DNA, then induce genetic mutations, which may induce tumor (1). At present, the interaction mechanism of pesticide and DNA has become a hot filed in environmental medicine and public health. Pesticides have cytotoxicity and genotoxicity in human, animals and plants (14). As reported, organic phosphorus pesticide chlorpyrifos can induce apoptosis in human mononuclear cells. In addition to single and double strand breaks caused by phosphorus pesticide chlorpyrifos, methyl parathion and malathion also damage DNA by DNA-protein cross-linking. Organic phosphorus pesticides (acephate, methamidophos, chloramine phosphorus, malathion, malaoxon) can damage DNA, increase intracellular ROS and lipid peroxidation product malonaldehyde (MDA), but reduce the activity of superoxide dismutase (SOD), catalase (CAT), glutathione (GSH).

Oxidative stress induced DNA damage. More and more data support that the increasing rate of chronic disease may be related to the use of pesticides. As a result, intervention of exposure to pesticides residues and toxic chemicals plays an important role in environmental medicine and preventive medicine.

## Results

### The metabolic imbalance and accumulation of metabolites

Metabolism are chemical reactions during the process of material and energy exchange, which is vital to maintain the organism in a stable structure and adapt to environment. Metabolic imbalance is the imbalance between supply and demand in digestion, absorption and excretion, which manifested as one or more substances disorders, changing internal environment of cells and leading to chronic diseases. Metabolic diseases are the most common metabolic imbalance diseases, such as glycometabolism disorder caused diabetes, lipometabolism disorder caused hyperlipidemia and obesity, uric acid metabolism disorder caused gout, and so on (15). In fact, protein, fat, carbohydrates and other metabolic dysfunction are coexist in the same person and show a series of clinical-metabolic syndromes in metabolic diseases. Obesity, hyperlipidemia, hypertension and diabetes are closely related to metabolic diseases (16). Increasing of uric acid in body can lead to the metabolism disorder of glucose, lipids and amino acid. Water and sodium retention leading to electrolyte metabolic disorder and hypertension. Metabolic imbalance, obesity, hypertension, diabetes, dyslipidemia, all of them are independent and dangerous factors to cardio-cerebro-vascular diseases (17). Control of blood lipid, blood pressure and body weight can significantly reduce the incidence and mortality of coronary heart diseases and cardiovascular diseases (18, 19).

Metabolic disorders can increase the risk of chronic kidney disease by about 50% (20). Immune system diseases are also affected by metabolism. Autoimmune hypothyroidism patients have abnormal blood lipid, increased blood homocysteine and serum uric acid compare to non-autoimmune hypothyroidism patients. Citric acid, glutamine, acetoacetic acid, pyruvic acid, hydroxyl butyric acid, histidine, glutamic acid, creatinine, dimethylamine and acetone in autoimmune hepatitis (AIH), primary biliary cirrhosis, drugs induced liver injury, all can be used to sensitively and specifically diagnose AIH. Malignant tumor is also related to metabolic system. It is reported that risk of cancer can increase as high as 56% due to metabolic syndromes (21). Obesity, hyperinsulinemia, hypertension and lipid metabolism disorders are closely related to women’s endometrial cancer. Obesity, insulin resistance can increase the risk of liver cancer, a variety of metabolic imbalances can lead to the occurrence of rectal cancer (22).

So, intervention of metabolic imbalance and accumulation of metabolites is vital to prevention and control of many chronic diseases.

### Inhibition of immune function and the development of chronic diseases

Immune system is the defense system of body, which not only defense the invasion of bacteria, fungi, viruses and other harmful substances, but also protect the health of the body by removing aged, mutated, deteriorated and dead cells in body. Immune system includes immune organs, immune cells and immune molecules. Proper function of each part ensures the stability of immune function. With the increase in age, accumulation of environmental toxins, change of life style, mental stress, nutritional status and other factors, immune function of human body also changes, which in turn affect the occurrence and development of diseases. In the unstable plaques of atherosclerosis patients, immune cells like antigen are aggregated presenting cells including mononuclear macrophage and dendritic cells (23, 24). In the process of cell activation, both antigen presenting cells and Th1 cells produce large number of inflammatory cytokines which significantly promotes atherosclerosis, destructs collagen, induces plaque ruptures, promotes the proliferation of smooth muscle cells, and induces secretion of IgG autoantibodies in B cells. These IgG autoantibodies can promote the incidence of atherosclerosis (23, 24). Alteration of immune function was not only related to the occurrence of cardiovascular and cerebrovascular diseases, but also related to multiple chronic diseases (25, 26). Mutations in human immune cells, immune function are related to tumor occurrence. Large number of immune suppression factors were found in the serum of tumor patients. Immune function in tumor patient is suppressed and related to the growth, relapse and metastasis of tumor (27, 28). Tumor cells induce weak antigen presentation, secrete immunosuppressive factors to avoid immune surveillance, while both are important mechanisms for tumor occurrence and development (29, 30). Relieving immune suppression, improve immune function will surely become the substantial section in prevention and intervention of cancer.

### Chronic diseases and chronic inflammatory factors

Chronic inflammation factors are cytokines associated with inflammation, which are produced and secreted by immune and few non-immune cells. They are involved in processes of cell growth, differentiation, repair and immune response (31, 32). Chronic inflammatory involved in the pathophysiology of most chronic diseases (33, 34). TNF-α can block insulin signal transduction in skeletal muscle by JUN amino terminal kinase (JNK), eventually affect glycometabolism, which is associated with the incidence of diabetes (35, 36). Content of plasma IL-6 in diabetic was significantly correlated with the content of homocysteine, which also is an independent risk factor of atherosclerosis (37–39).

Occurrence of many tumors are caused by long-term stimulation of certain chronic inflammatory. Risk of intestinal cancer greatly increased in patients with chronic ulcerative colitis. Researches showed that serum level TNF-α, IL-lα and IL-1β in liver cancer patients were significantly higher, especially higher in patients with recurrence of liver cancer (40, 41). Application of inflammatory enzyme Cox-2 inhibitor celecoxib with 5-FU or radiotherapy respectively can significantly reduce survival ability of tumor cells (42). IL-1β, TNF-α, IL-8 and other inflammatory factors influence prognosis by increasing local inflammatory reaction after surgery or chemotherapy (43).

Many scholars support “hypothesis of endothelial injury” in the pathogenesis of atherosclerosis, they think that formation of plaques is the result of inflammation and hyperplastic fiber reaction induced by endothelium and tunica intima injuries. Macrophage accumulation, oxidation of LDL-C, secretion of IL-1β, TNF-α and other inflammatory factors would promote formation of tumor plaques and inflammatory reactions (44–46). In addition, it was found that level of TNF-α in plasma of hypertension patients was significantly higher. The reason might be that TNF-α affect proliferation, differentiation and regulation of vascular smooth muscle cells to thicken the vessel wall, stenosis severity of lumen, increase peripheral resistance, and then increase blood pressure (47). In a word, prevention and intervention chronic irritation and injury of chronic inflammatory factors is very important for prevention and intervention of chronic diseases.

### Protein instability and cell function

Proteins are important component of human cells and tissues, accounting for 16%–20% of body weight. Proteins involved in gene expression, physiological regulation, maintenance of metabolism, regulate almost all biological functions in cell and cell matrix. When proteins are exposed to heavy metals, pesticides, physical, chemical and biological damage, protein conformation will be abnormal or misfolded, causing loss of biological function and corresponding diseases. Abnormal protein structure is closely related to nervous system diseases. In Alzheimer’s disease (AD) patients, abnormal folding and deposition of beta amyloid protein leads to neuritic plaque in brain. One important pathological basis of AD is misfolding and aggregation causing entanglement of hyper-phosphorylated microtubule associated protein TAU (48). Louis’s body of Parkinson’s disease is fibrous structure comes from the abnormal beta folding of unfolded α-synuclein (49). Huntington’s disease is caused by extension of huntingtin’s N-terminal poly-valley amide which changes protein conformation into β-pleated sheet (50).

Structural changes in protein also increase the risk of cardiovascular diseases. Lipoproteins are closely related to cardiovascular diseases. In a normal body, different lipid proteins perform their functions to maintain homeostasis. When oxidative or glycosylation modification happened to low density lipoprotein (LDL), it stimulates, secretes a variety of growth factors and pro-inflammatory mediators, accelerates differentiation rate of monocytes, promotes atherosclerotic, changes lipid stripes into fibrous plaques. High density lipoprotein (HDL) oxidation will damage biological function, decrease reverse cholesterol transport, which not only lost its protective function but also causes cell toxicity, decreases fibrinolytic activity, increases coagulative activity, then lead to thrombosis and significantly higher incidence of cardiovascular diseases.

Metabolic diseases are also associated with protein conformation alternation. In normal circumstances, protein acetylation and deacetylation have synergetic effect. Once the balance is disturbed, gene expression regulation will be in disorder, which can easily lead to related diseases. It was found that there was a large number of acetylated metabolism enzymes in cytoplasm and mitochondria. Loss of the acetylation sites can result in the changes of the enzyme activity and induce metabolic disorders (51, 52). Diabetes, obesity and other diseases may be associated with mutation of acetylation sites in metabolic enzymes. Modification of histone proteins was also closely related to glucose metabolism.

Stability of protein molecules not only causes the above diseases, but also plays important roles in the development of malignant tumors. Methylation of histone is closely related to breast cancer, colon cancer, liver cancer (53). Histone demethylation plays an important role in the regulation of tumor microenvironment (54). In plasma membrane of breast cancer cells, 25 proteins get 27 N-glycosylation sites, such as *BRCA-1, CD44, EGFR*, can influence invasion and metastasis of breast cancer by regulating differentiation and proliferation of tumor cells. High expression of phosphorylated tyrosine was related to cell activity, migration and anti-apoptosis in metastatic hepatocarcinoma cells (55, 56).

Proteins have high efficiency and specificity functions. They can adapt their structure and function to exogenous stimulations. With the increase in age, protective effect of anti-free radicals decreased, function specificity and structure stability of proteins begin to change. Aging related proteins mainly show covalent changes (oxidation, glycosylation, deamidation, etc.) and conformational change (57, 58). Covalent change is a permanent irreversible damage because of the characteristic change of amino acids. Conformational change can be eliminated by unfolding and folding the protein. Misfolded and aggregated amyloid protein can promote the development of Creutzfeldt-Jakob disease (59). So, maintaining the protein stability is one of the important aspects to prevent the chronic diseases.

### Gut Microbiota and the development of chronic diseases

The gut microbiota exists in a state of “normobiosis” in which microorganisms with beneficial effects on health predominate over harmful species. The gut microbiota regulates many physiological functions, ranging from energy regulation and cognitive processes to toxin neutralization and immunity against pathogens (60, 61). The gut microbiota appears to be critical to maintain host homeostasis and health, however, the alterations in the composition of the gut microbiota have been shown to contribute to the development of various chronic diseases (62). Inability to regulate intestinal mucosal immunity can result in local and systemic inflammation (63). Gut microorganisms may stimulate production of pro-inflammatory cytokines and infiltration of immune cells. A persistent low level of inflammation in different organs also contributes to diabetes, heart disease, and obesity (64).

Genetic and environmental factors influence the abundance and type of beneficial and pathogenic bacteria in the gut, with each type of bacteria possibly having preferred substrates for growth and producing unique fermentation products. Such fermentation end products may also influence food intake, energy levels, and insulin activity, thereby influencing adiposity and related metabolic pathways. “Proteins and peptides reaching the colon are fermented by intestinal bacteria to yield a great diversity of end products, including branched-chain fatty acids, such as isobutyrate and isovalerate, along with ammonia, amines, N-nitroso compounds, phenols, in doles, thiols, CO2, H2, and sulfur-containing compounds such as H2S, many of which have toxic properties (65) that have been associated with colon cancer (66) and inflammatory bowel disease” (67).

The gut microbiota is involved in the production of metabolites [trimethylamine (TMA) and trimethylamine N-oxide (TMAO)] that increase the risk of cardiovascular disease (68, 69). The digestion of red meat by the gut microbiota is also associated with increased risk of colorectal cancer. In addition to the compounds found in meat (e.g. proteins, heme) and the compounds generated by the cooking process (e.g. N-nitroso compounds, heterocyclic amines), increased bacterial fermentation (putrefaction) of undigested proteins and production of bacterial metabolites derived from amino acids may affect the functions and renewal of epithelial cells lining the colon (70).

A complex relationship exists between diet, microbes, and the gut epithelium. During “dysbiosis,” in which a few potentially harmful bacterial genera or species have been shown to propagate, a disease-prone situation is created. Diet-induced dysbiosis was identified as a contributing factor for the development of diseases such as allergy, autoimmune disease, Crohn’s disease, obesity, type-2 diabetes, and ulcerative colitis, irritable bowel syndrome, and NAFLD (61, 71). In addition, intestinal microbes may contribute to obesity by inducing chronic inflammation (72). In summary, the human intestinal microbiota is similar to a true organ, which plays critical roles in human health and disease.

### Molecular criteria for the use of TE-PEMIC in prevention of chronic diseases

Large number of people have the risk of chronic diseases and the number increases every year. It has become the world’s top public health issue. How to effectively contain chronic diseases and decrease the ratio of high risk population have become the most urgent task. In the traditional three level prevention and control system, it has achieved good chronic diseases suppress effect by advertisement, education, nutrition, sports and lifestyle intervention. But current clinical care is mainly concerned with the two level prevention and control of chronic diseases, which is carrying out early diagnosis and early treatment of specific chronic diseases. TE-PEMIC chronic diseases prevention and control system integrates environmental medicine, functional medicine, molecular medicine, cellular immunology and the latest biomedical technology, and it provide each guest with individualized health and diseases risk assessment, determine whether the disease risk exist by laboratory inspections. Is it suitable for prevention and treatment of chronic diseases by TE-PEMIC chronic diseases prevention and control system?

Laboratory test items are as follows:

Early molecular warning: using genomics technology, detection and analysis of the molecular genetic risk of diseases, to give early warning of chronic diseases.
Analysis of protein instability: Using affinity purification and proteomics technology, analyze structural instability and biological function of major biomacromolecules (such as albumin, lipoprotein, immune globulin, etc.)
Environmental toxin residues and exposure index analysis: Using environmental genomics technology to provide customers with individualized risk analysis of environmental toxins, using environmental medicine to analysis pathogenic heavy metals, pesticides, insecticides, chemicals and exposure index in vivo.
Endogenous metabolic accumulated toxins: Using functional medicine and clinical biochemistry technology to assess nucleic acids, proteins, lipids and sugar metabolism and detect changes of metabolic related products, such as HCY, free radicals, etc.
Immune inhibitor detection: Using flow cytometry and immunological techniques to evaluate function of cellular immunity and humoral immune, monitor changes of immune complex and autoimmune diseases related indicators.
Chronic inflammatory factors: monitoring TNF-α, IL-1β, IL-6, IL-8

## Discussion

### TE-PEMIC chronic diseases prevention and control system

Toward chronic diseases prevention and control to earlier stage is an important national health strategy. At the same time, transition to the management of high-risk population and risk intervention is the technical difficulty of health industry. TE-PEMIC chronic disease prevention and control system will take unstable proteins, environmental toxins residue, accumulated endogenous metabolic toxins, immunosuppressive factors, chronic inflammatory factors and gut microbiota and its dysbiosis as important aspects of exploration and key issues to be resolved. According to in vivo diseased risk assessment results, using international advanced dual-mode target blood filtration technology can achieve rapid intervention of chronic diseases, control high risk individuals’ risk, give them an opportunity to restore balance. In addition to the use of continuous dual-mode targeted blood filtration to remove risk factors in blood and tissue, TE-PEMIC chronic diseases prevention and control system can also target each risk factor to build individual molecular monitoring and health warning mechanism, combine with dietetic regulation which based on analysis of intestinal flora diversity, nutritional intervention to meet metabolic balance, management and intervention of individual life for the purpose of the long-term risk control. Targeted intervention of preventive medical care will reduce the risk of diseases.

## Conclusion

The initial establishment of TE-PEMIC chronic diseases prevention and control system is an innovative exploration of preventive medical technology and service. It is also a new attempt to standardization of preventive medical technology and service. Although we have got some clinical results recently, there are still much to do in the future, including continuously improve the theory, technology, clinical evaluation, long-term prevention and so on.

## Ethical considerations

Ethical issues (Including plagiarism, informed consent, misconduct, data fabrication and/or falsification, double publication and/or submission, redundancy, etc.) have been completely observed by the authors.
